# How to handle lipemic CBC samples on Sysmex hematology analyzers?

**DOI:** 10.1515/almed-2024-0206

**Published:** 2025-03-20

**Authors:** Vanja Radišić Biljak, Lucija Dolovčak, Iva Bakarić, Ana Nikler, Andrea Saračević, Marija Grdić Rajković

**Affiliations:** Department of Medical Laboratory Diagnostics, University Hospital “Sveti Duh”, Zagreb, Croatia; Department of Sport and Exercise Medicine, Faculty of Kinesiology, University of Zagreb, Zagreb, Croatia; Department of Medical Biochemistry and Hematology, Faculty of Pharmacy and Biochemistry, University of Zagreb, Zagreb, Croatia

**Keywords:** complete blood count (CBC), hemoglobin, lipemia, interference

## Abstract

**Objectives:**

Lipemia poses a significant preanalytical problem for complete blood count (CBC) measurement due to limited and non-standardized methods for recognition and removal. We aimed to verify the optical hemoglobin (Hb-O) measurements on the Sysmex XN-1000 hematology analyzer (HA) as a possible reliable method for managing lipemic CBC samples.

**Methods:**

Ninety CBC samples with varying Hb concentrations were gradually spiked with a lipid emulsion. Measurements were repeated and Hb-O concentrations were recorded. Spiked CBC samples were centrifuged (400 *g*/10 min). Plasma was carefully removed, and Hb concentration was measured. The values obtained from the lipemic samples were adjusted according to the measurements in the plasma. The removed plasma was substituted with the analyzer’s diluent, and measurements were repeated. Triglyceride concentrations were measured in lipemic plasma samples.

**Results:**

Hb-O showed statistically insignificant and acceptable bias compared to the initial Hb measurement according to the strictest acceptability criteria (−0.4 %, 95 % CI: −1.2–0.3, p=0.2447). The observed bias did not correlate with the degree of lipemia (rho=−0.072, 95 % CI: −0.295 to 0.157, p=0.537). Hemoglobin measured in samples with lipemic plasma replaced by analyzer diluent exhibited minimal, albeit statistically significant, bias (−1.1 %, 95 % CI: −2.0 to (−0.1), p=0.025). The observed bias negatively correlated with the degree of lipemia (rho=−0.369, 95 % CI: −0.550 to (−0.155), p=0.001). The highest unacceptable bias was found in the recalculated hemoglobin values based on the measured plasma hemoglobin (−3.5 %, 95 % CI: −4.1 to (−2.9), p<0.0001).

**Conclusions:**

Hb-O measurement is the most reliable measure of lipemia removal in CBC samples on the Sysmex XN-1000 HA.

## Introduction

Lipemia, defined as sample turbidity due to the accumulation of lipoprotein particles, causes a significant problem in the clinical laboratory setting [1]. Although relatively uncommon, it poses a significant preanalytical problem for complete blood count (CBC) measurement in lipemic whole blood samples for various reasons. As most hematology analyzers (HA) measure hemoglobin spectrophotometrically on 530 nm, lipemia interferes with hemoglobin measurement, causing falsely high concentrations of hemoglobin and, consequently, mean corpuscular hemoglobin (MCH) and mean corpuscular hemoglobin concentration (MCHC) [2]. In biochemistry and endocrinology measurements hemolysis, icteria and lipemia interference is mostly identified visual inspection, by (semi)quantitative HIL measurements, or some combination of both [3]. Contrary, the most common alert that could indicate a lipemic CBC sample is the increase of MCHC above a certain threshold. Although some authors propose MCHC of 360 g/L as a lipemia threshold [4], every laboratory should have an established cut-off level of lipemia above which results should not be reported [5]. Several possible solutions to eliminate lipemia interference in CBC measurement are present in routine hematology laboratories worldwide [6]. One includes hemoglobin concentration measurement in spun lipemic plasma samples. The obtained hemoglobin value measured in lipemic plasma samples allows further whole blood hemoglobin concentration correction. The measured hemoglobin concentration from the lipemic plasma sample is subtracted from the hemoglobin concentration in the whole blood sample. After correction, MCH and MCHC values are recalculated. Another widespread lipemia removal procedure is the plasma replacement procedure. This procedure involves removing the lipemic plasma and replacing it with an appropriate volume of the analyzer’s diluent, followed by a sample re-analysis. The most practical solution for obtaining reliable CBC results from lipemic whole blood samples is employing hematology analyzers insensitive to lipemia interference. Siemens Advia 2120i HA measures cellular hemoglobin concentration mean (CHCM) – hemoglobin is measured directly in the intact erythrocytes. This approach allows for accurate hemoglobin measurement without interference from lipemia [7], [8], [9].

In 2019 The Croatian Working Group for Laboratory Hematology, on behalf of the Croatian Society of Medical Biochemistry and Laboratory Medicine, wanted to explore the background in field of laboratory hematology routine practice among Croatian laboratories in order to develop future strategies for producing national recommendations in order to promote harmonization in the field [5]. The survey showed various strategies for resolving lipemia-related issues in CBC measurements [5]. The most common strategy was the first procedure in which hemoglobin concentration was measured in spun lipemic plasma samples, which accounted for 40 % of cases [5]. However, this method requires considerable sample manipulation and necessitates recalculating results to ensure reliable hemoglobin values.

Until recently, our laboratory utilized Siemens Advia 2120i hematology analyzers. Thus, lipemia certainly was not an issue for CBC measurements. However, Advia has been replaced with Sysmex XN-1000. Therefore, in order to optimize and standardize our practice, as well as to ensure patient safety, we aimed to establish our lipemia interference protocol for reliable hemoglobin measurement. Although Sysmex recognizes this possible sample interference, the company does not provide any practical solution for lipemia removal under the System limitations subheading in the Automated Hematology Analyzer XN series (XN-1000) Instructions for Use user manual. However, based on the recent Berda-Haddad et al. study results [10], Sysmex Corporation proposes an algorithm for resolving increased MCHC measurements. This algorithm can be integrated into laboratory-extended IPU (EPU) if available. As increased sample turbidity is one of the possible causes of increased MCHC measurements, based on the proposed Sysmex algorithm, a reliable method for eliminating lipemia interference in lipemic CBC samples is the measurement of optical hemoglobin in the reticulocyte measurement mode (Hb-O) [11]. However, Hb-O is currently a research-only parameter and is not included in the quality control (QC) menu. Therefore, we wanted to explore the analytical characteristics of the new optical hemoglobin measurement and compare it with the most commonly used lipemia-eliminating protocols.

## Materials and methods

The experimental study was performed in the Department of Medical Laboratory Diagnostics, University Hospital “Sveti Duh”, Zagreb, Croatia, during October and November 2024.

### Precision

Before the lipemia assessment, a short precision study was performed regarding Hb-O measurements on the Sysmex XN-1000 hematology analyzer (HA) (Sysmex, Kobe, Japan). Within-day precision was explored by using patient samples in three different concentration ranges, and commercial control samples (Sysmex XN Check, lot: Level 1 42461101, Level 2 42461102, Level 3 42461103, exp: 24.11.2024.) by 20 consecutive analyses. Between-day precision was established by using commercial control samples, daily for 30 days. The coefficient of variation was calculated for within- and between-day precision study.

### Accuracy

Ninety whole blood K_2_EDTA patient samples with varying Hb concentrations and a clinician request for CBC were analyzed on Sysmex XN-1000 HA. The initial CBC measurement was considered the true value. To ensure various Hb concentrations, samples were selected according to the distribution presented in Table 1.

**Table 1:** The distribution of required whole-blood K_2_EDTA samples for the lipemia removement verification protocol.

Initial Hb concentration, g/L	Addition of lipid emulsion, µL					
	20	40	60	80	100	120
50–110	5	5	5	5	5	5
110–150	5	5	5	5	5	5
>150	5	5	5	5	5	5

To simulate lipemia interference the samples were gradually spiked with a SMOFlipid 200 mg/mL Emulsion zur Infusion lipid emulsion (Fresenius Kabi, Graz, Austria, exp. 03/2025), by following the predetermined emulsion volumes (Table 1).

After the addition of lipemic emulsion CBC measurements were repeated, and standard Hb concentrations measured by spectrophotometrical method were recorded.

Verification protocol for reliable measurement of CBC parameters in lipemic whole blood samples on the Sysmex XN-1000 hematology analyzer included three different methods:

1. Measurement of lipemic samples in reticulocyte measurement mode to obtain Hb-O and MCHC-O measurements.
2. Hemoglobin concentration measurement in spun lipemic plasma samples.
  Spiked lipemic samples were centrifuged on 400 *g* for 10 min. Lipemic plasma was carefully removed in order not to disturb the cells, and standard Hb concentrations were measured in lipemic plasma samples. Consenquently, the values obtained from the lipemic samples were adjusted according to the measurements in the plasma by following the simple equation:
$${\text{Hb}}_{\text{corrected}}\;\left(g/L\right)={\text{Hb}}_{\text{lipemic}\;\text{sample}}\;–\;\left[{\text{Hb}}_{\text{lipemic}\;\text{plasma}}\times \left(1\;–\text{ hematocrit }\left(L/L\right)\right)\right]\text{.}$$
  MCH and MCHC were calculated as instructed:
$$\text{MCH}=\frac{\text{Hb}\left(g/L\right)}{\text{Erc}\;\left(\times 10^{12}/L\right)}\left(pg\right)$$
$$\text{MCHC}=\frac{\text{Hb}\left(g/L\right)}{\text{Hct}\;\left(L/L\right)}\left(g/L\right)$$
3. Replacement of the removed lipemic plasma with an adequate volume of the analyzer’s diluent.
  The removed plasma was substituted with the analyzer’s diluent (Sysmex Cellpack™ DCL). After thorough mixing, measurements were repeated and standard Hb concentrations were recorded.

To assess the degree of simulated lipemia, triglyceride concentrations, as well as HIL index, were measured in lipemic plasma samples on Abbott Alinity c analyzer (Abbott, Abbott Park, IL, USA). Tryglicerides were measured utilizing the glycerol phosphate oxidase method that produces a quinoneimine dye, where the absorbance of the dye, measured at 604 nm is proportional to the concentration of triglyceride present in the sample. HIL indices were measured spectrophotometrically using different wavelength pairs and a specific algorithm to obtain HIL index values that approximately correspond to free hemoglobin, bilirubin and triglycerides concentrations.

The whole protocol is presented in Table 2.

**Table 2:** Verification protocol for reliable measurement of CBC parameters in lipemic whole blood K_2_EDTA samples on the Sysmex XN-1000 hematology analyzer.

Steps	Analysis	Additional
CBC measurement in non-lipemic native samples	Hemoglobin, MCH and MCHC measured in the native samples will be considered as “true value”.	Print the original CBC measurement.
Lipid emulsion addition	Smoflipid lipid emulsion will be added to the selected samples. Starting with 20 µL and gradually increasing the volume.	The samples will be gently mixed well to evenly distribute the lipid emulsion.
Hb-O and MCHC-O measurement	Lipemic samples will be analyzed in the retic measurement mode.	The Hb-O and MCHC-O measurement is located in the Lab only tab.
Lipemic sample centrifugation	Lipemic samples will be centrifuged for 10 min at 400 *g*.	Choose CSF program on the centrifuge.
Lipemic plasma removal	After centrifugation, the plasma level in the CBC sample will be recorded.	The lipemic plasma will be carefully separated into a clean plastic tube using an automatic pipette.
Hb measurement in lipemic plasma samples	Separated lipemic plasma will be analyzed in open sampling mode on a Sysmex XN-1000 analyzer.	The result of Hb in the lipemic plasma is printed. Plasma is referred to biochemistry for triglyceride and HIL index measurement.
Addition of analyzer’s diluent	Analyzer diluent (Sysmex Cellpack™ DCL) will be added to the CBC sample up to the mark where the plasma has reached.	The sample will be gently mixed well to evenly distribute the diluent throughout the sample.
Reanalyzing the sample	The CBC samples will be reanalyzed on Sysmex XN-1000 HA.	The results are printed.
Results entry	All results are entered in the suitable Excel sheet.	All possible problems during the implementation of the protocol are recorded.

No patient information was collected in this study as the interference study included the remnant plasma samples with CBC orders. The ethics committee ot University Hospital “Sveti Duh”, Zagreb, Croatia granted an universal approval for verification studies on remnant samples. They confirmed that patient consent in such studies were not needed.

### Statistical analysis

An average bias between the measurements obtained from different lipemia removing methods, compared to the initial measurements, was calculated by employing the Bland-Altman plot. Average bias was expressed as percentage and compared to biological variability criteria for bias according to the European Federation of Clinical Chemistry and Laboratory Medicine (EFLM) Biological Variation Database [12]. To assess the possible correlation of the calculated biases and the degree of lipemia, as well as hemoglobin concentration, rank correlation was performed [13]. Differences between three lipemia removing methods were tested by the Friedman test, as variables were not normally distributed. p values<0.05 were considered statistically significant. Statistical analysis was performed in MedCalc^®^ Statistical Software version 23.0.2 (MedCalc Software Ltd, Ostend, Belgium).

## Results

### Precision

The precision study results were within the acceptance criteria and are presented in Table 3. The Hb-O measurement on Sysmex XN-1000 HA is currently a research-only parameter, and there are no manufacturer-defined acceptability criteria for precision. Therefore, we have used the biological variability criteria for precision.

**Table 3:** Results of the precision study for measurement of optical hemoglobin on Sysmex XN-1000 hematology analyzer.

Precision, %	Patient samples									Control samples								
	Low			Medium			High			L1			L2			L3		
	$\bar{x}$	CV	BV	$\bar{x}$	CV	BV	$\bar{x}$	CV	BV	$\bar{x}$	CV	BV	$\bar{x}$	CV	BV	$\bar{x}$	CV	BV
HB-O	101	1.4	2.0	119	1.6	2.0	141	1.3	2.0	56	1.7	2.0	104	0.9	2.0	132	1.7	2.0

HB-O, measurement of optical hemoglobin on Sysmex XN-1000 hematology analyzer; CV, coefficient of variation; BV, acceptance criteria for precision derived from the EFLM biological variability database (miminum).																		
	Within acceptance criteria																	

### Accuracy

Fifteen samples with the highest degree of simulated lipemia (addition of 120 μL of lipid emulsion) were grossly hemolyzed and were excluded from further statistical analysis. The gradual increase in hemoglobin concentration, MCHC measurement and triglyceride concentration is presented in Table 4.

**Table 4:** The gradual increase of hemoglobin and MCHC concentration after addition of lipid emulsion.

Added volume of lipid emulsion		Hb native	Hb+ lipid emulsion	Percentage change	MCHC native	MCHC+ lipid emulsion	Percentage change	TG	L index	H index
20	Median	113	117	3.5	336	350	4.1	8.2	264	31
	IQR	97–166	103–170	2.9–5.5	331–341	339–356	3.0–5.8	7.2–9.8	223–387	11–64
40	Median	115	124	7.1	333	361	9.1	14.7	497	19
	IQR	97–156	107–162	4.8–9.7	328–339	352–373	6.8–11.7	12.8–18.6	454–622	5–42
60	Median	138	148	10.6	331	369	13.1	21.6	803	66
	IQR	90–158	103–173	7.5–14.3	323–340	362–400	9.5–17.8	16.9–30.3	647–1115	27–92
80	Median	127	148	13.9	330	381	15.8	25.7	998	61
	IQR	102–156	117–168	11.2–15.1	318–341	369–399	13.9–18.5	22.8–33.4	797–1181	16–91
100	Median	134	158	17.9	332	404	21.4	40.5	1539	37
	IQR	94–157	125–176	12.8–27.0	314–341	389–438	13.8–36.3	32.7–45.7	1192–1712	22–94

Reference change value (RCV) for hemoglobin=6.8 %; RCV for MCHC=3.0 %.										
	Within RCV						Greater than RCV			

The results of the accuracy study are presented in Table 5. Hb-O showed statistically insignificant and acceptable bias compared to the initial Hb measurement according to the strictest acceptability criteria (−0.4 %, 95 % CI: −1.2–0.3, p=0.2447). Hemoglobin measured in samples with lipemic plasma replaced by analyzer diluent exhibited minimal, albeit statistically significant, bias (−1.1 %, 95 % CI: −2.0 – (−0.1), p=0.025). The highest unacceptable bias was found in the recalculated hemoglobin values based on the measured plasma hemoglobin (−3.5 %, 95 % CI: −4.1 – (−2.9), p<0.0001).

**Table 5:** Comparison of hemoglobin, MCH and MCHC concentrations in lipemia removal methods.

Lipemia removal method		Bias %	95 % CI	p-Value	Biological variability criteria for bias^a^		
					Optimal bias	Desirable bias	Minimum bias
1. Measurement of lipemic samples in reticulocyte measurement mode							
Hb-O		−0.4	−1.2 to 0.3	0.244	0.8	1.7	2.5
MCH (Hb-O/RBC)		−1.2	/	/	0.6	1.2	1.7
MCHC-O		1.3	/	/	0.2	0.4	0.6
MCHC (Hb-O/HCT)		−0.5	/	/	0.2	0.4	0.6
2. Hemoglobin concentration measurement in spun lipemic plasma samples							
Hb corrected		−3.5	−4.1 to (−2.9)	<0.001	0.8	1.7	2.5
MCH calculated		−4.4	/	/	0.6	1.2	1.7
MCHC calculated		−1.5	/	/	0.2	0.4	0.6
3. Replacement of the removed lipemic plasma with the analyzer’s diluent							
Hb diluent		−1.1	−2.0 to (−0.1)	0.025	0.8	1.7	2.5
MCH diluent		2.5	/	/	0.6	1.2	1.7
MCHC diluent		2.7	/	/	0.2	0.4	0.6
MCHC (Hb diluent/HCT)		0.7	/	/	0.2	0.4	0.6

^a^Aarsand AK, Fernandez-Calle P, Webster C, Coskun A, Gonzales-Lao E, Diaz-Garzon J, et al. The EFLM Biological Variation Database. https://biologicalvariation.eu/. Allowed bias: <0.25 × (CVI^2^ + CVG^2^)^1/2^ The factor 0.25 refers to desirable Analytical Performance Specification (APS). The factor for optimum and minimum performance specifications are 0.125 and 0.375, respectively. CVI, within-subject (CVI) estimate; CVG, between-subject (CVG) estimate. Available at: Aarsand AK, Fernandez-Calle P, Webster C, Coskun A, Gonzales-Lao E, Diaz-Garzon J, et al. The EFLM Biological Variation Database. https://biologicalvariation.eu/. Hb-O, measurement of optical hemoglobin on Sysmex XN-1000 hematology analyzer; MCHC-O, measurement of optical MCHC concentration on Sysmex XN-1000 hematology analyzer; RBC, red blood cell concentration; HCT, hematocrit.							
	Within acceptance criteria				Greater than acceptance criteria		

MCHC measured with all three lipemia removal protocols showed unacceptable results according to the acceptability criteria for accuracy. However, if a mathematical correction is applied to the calculation of MCHC by using the Hb-O measurement, we obtained an acceptable bias according to the criteria of minimally acceptable accuracy compared to native MCHC.

Friedman’s test showed a statistically significant difference between the observed hemoglobin measurements (p<0.00001). Post hoc testing showed a statistically significant difference between hemoglobin measured in the native sample and hemoglobin obtained by correction for plasma hemoglobin and hemoglobin measured in samples where plasma was replaced by the analyzer’s diluent (p<0.05). No statistically significant difference was observed between initial hemoglobin measurement and Hb-O (Table 6). The same results were obtained when all included hemoglobin measurements were divided into categories: <110 g/L, 110–150 g/L, and >150 g/L, thus reflecting low, normal, and high hemoglobin concentrations. Hb-O measurements were equally reliable independently of concentration spans (Table 6).

**Table 6:** Comparison of measurements by using the Friedman statistical test for paired samples.

Comparison of measurements, n=75	1. Native sample measurement	2. Optical measurement	3. Spun lipemic plasma samples correction	4. Replacement of the removed lipemic plasma with the analyzer’s diluent	p-Value
	Median (IQR)	Median (IQR)	Median (IQR)	median (IQR)	
Hemoglobin (all samples)	132 (101–158)	129 (102–158)	124^a^ (97–151)	126^a^ (98–155)	<0.00001
<110 g/L	92 (83–98)	91 (83–96)	89^a^ (83–96)	92 (86–98)	0.00484
110–150 g/L	128 (119–137)	127 (121–137)	122^a^ (116–131)	125^a^ (119–133)	0.00001
>150 g/L	162 (158–167)	163 (158–170)	158^a^ (155–167)	155^a^ (151–164)	<0.00001
MCH	30.2 (28.4–31.2)	30.2 (28.3–31.2)	29.2^a^ (27.5–30.1)	30.9^a^ (29.3–32.6)	<0.00001
MCHC	334 (323–340)	340^a^ (324–353)	331^a^ (319–337)	341^a^ (331–348)	<0.00001

^a^ *Post-hoc* statistically significant difference compared to the initial measurement in the native sample (p<0.05).

The rank correlation statistical analysis explored the possible correlations between the observed biases in Hb measurement in all three lipemia removal methods and the degree of lipemia/hemolysis (measured by triglyceride concentration and HIL indexes). The results are presented in Table 7.

**Table 7:** Correlation between deviations in hemoglobin measurement in lipemic samples and the degree of lipemia and hemolysis.

Calculated bias	Triglycerides (mmol/L)		Lipemia degree (L index)		Hemolysis (H index)	
	Rho (95% CI)	p-Value	Rho (95% CI)	p-Value	Rho (95% CI)	p-Value
Hb-O	−0.072 (−0.295 to 0.157)	0.537	−0.041 (−0.266 to 0.187)	0.725	0.068 (−0.161 to 0.291)	0.559
Hb-corrected	−0.915 (−0.946 to (−0.868))	<0.001	−0.918 (−0.947 to (−0.873))	<0.001	−0.390 (−0.566 to (−0.178))	0.001
Hb-diluent	−0.369 (−0.550 to (−0.155))	0.001	−0.412 (−0.590 to (−0.196))	0.001	−0.406 (−0.585 to (−0.190))	0.001

	Statistically significant correlation.					

## Discussion

The Sysmex XN-1000 Hb-O measurement has effectively addressed interference caused by lipemia in lipemic whole blood EDTA samples. By re-analyzing samples in the reticulocyte measurement mode, we eliminated the need for the manual procedures traditionally used in medical-biochemistry laboratories to remove lipemia interference.

While we observed a significant increase in hemoglobin (Hb) concentrations, even with the smallest addition of lipids (20 µL), this increase was not clinically significant when compared to reference change values (RCV) obtained from the EFLM database. The concentration of triglycerides in such samples could reach up to 10 mmol/L. However, further addition of lipid emulsion did result in Hb concentrations exceeding the clinically significant RCV, prompting us to reevaluate the proposed Sysmex MCHC cut-off of 365 g/L as an indicator of lipemic CBCs. To minimize unnecessary testing and, at the same time effectively identify lipemia interference, we suggest a lower MCHC threshold of 360 g/L that could enhance the management of analytical interferences, similar to recommendations made by Henry et al. [14].

Another advantage of Hb-O measurement, in contrast to the existing protocols for lipemia removal, is its independence from the levels of both lipemia and hemolysis. It is well-known that increased lipid concentration is associated with heightened hemolysis, as demonstrated in our study [15]. The typical approach for addressing lipemia-related issues in CBC measurements in Croatia – measuring hemoglobin concentration in spun lipemic plasma samples – is significantly affected by both the degree of lipemia and hemolysis, leading to unreliable results in samples with high levels of lipemia. Similar findings were reported in a study by Aruga et al., which highlighted the effectiveness of the optical method for measuring hemoglobin in chylous samples [16]; however, their research did not include a comparison with the commonly used lipemia-removal methods.

Our study does have some limitations. The results might vary with different lipid emulsions used for simulating lipemia interference. However, simulating lipemia this way is the best we can achieve in laboratories. The absence of internal quality control or Hb-O measurement presents a potential concern regarding using this research-only parameter in routine clinical assessments. Although Hb-O showed higher total imprecision compared to the spectrophotometrically measured Hb concentrations, the observed imprecision was well within the Sysmex acceptance criteria. Sysmex internal QC samples can further be validated for Hb-O measurement in various QC lots.

Despite some shortcomings, we have successfully demonstrated that Hb-O measurement using the Sysmex HA is superior in eliminating lipemia interference compared to existing lipemia removal methods used worldwide, including in Croatia. This method offers a straightforward approach to standardizing the lipemia elimination protocol for complete blood count (CBC) measurements, without necessitating any manipulation of the samples. Traditional lipemia removal techniques often involve sample manipulation, which can introduce significant bias, potentially leading to erroneous results and compromising patient safety [17], 18]. Ultimately, adopting a simple and safe procedure for lipemia removal through direct Hb-O measurement could reduce the overall workload in light of the globally recognized shortage of laboratory personnel [19].
